# Management of diastasis of the rectus abdominis muscles: recommendations for swedish national guidelines

**DOI:** 10.1177/1457496920961000

**Published:** 2020-09-28

**Authors:** Anders Carlstedt, Sven Bringman, Mattias Egberth, Peter Emanuelsson, Anders Olsson, Ulf Petersson, Joakim Pålstedt, Gabriel Sandblom, Rune Sjödahl, Birgit Stark, Karin Strigård, Jael Tall, Elvar Theodorsson

**Affiliations:** Department of Surgery, Karlstad Central Hospital, Karlstad, Sweden; Department of Surgery, Södertälje Hospital, Stockholm, Sweden; Department of Clinical Sciences, Danderyds Hospital, Karolinska Institutet, Stockholm, Sweden; Department of Surgery, Mora hospital, Mora, Sweden; Department of Molecular Medicine and Surgery, Karolinska Institutet, Solna, Sweden; Department of Clinical Science and Education Södersjukhuset, Karolinska Institutet, Stockholm, Sweden; Clinic of Surgery, Capio CFTK, Stockholm, Sweden; Department of Surgery, Skåne University Hospital, Lund University, Malmö, Sweden; Department of Clinical Sciences, Danderyds Hospital, Karolinska Institutet, Stockholm, Sweden; Department of Surgery, Ersta Hospital, Stockholm, Sweden; Department of Surgery, Södersjukhuset, Stockholm, 118 83, Sweden. Department of Clinical Science and Education Södersjukhuset, Karolinska Institutet, Stockholm, Sweden; Department of Surgery, Linköping University Hospital, Linköping, Sweden; Department of Molecular Medicine and Surgery, Karolinska Institute, Solna, Sweden; Department of Surgical and Perioperative Sciences, Umeå University, Umeå, Sweden; Department of Clinical Sciences, Danderyds Hospital, Karolinska Institutet, Stockholm, Sweden; Department of Surgery, Ersta Hospital, Stockholm, Sweden; Department of Clinical Chemistry and Department of Clinical and Experimental Medicine, Linköping University, Linköping, Sweden

**Keywords:** Diastasis of the rectus abdominis muscles, guidelines, linea alba, pregnancy, physiotherapy, mesh

## Abstract

**Background:**

Diastasis of the rectus abdominis muscle is a common condition. There are no generally accepted criteria for diagnosis or treatment of diastasis of the rectus abdominis muscle, which causes uncertainty for the patient and healthcare providers alike.

**Methods:**

The consensus document was created by a group of Swedish surgeons and based on a structured literature review and practical experience.

**Results:**

The proposed criteria for diagnosis and treatment of diastasis of the rectus abdominis muscle are as follows: (1) Diastasis diagnosed at clinical examination using a caliper or ruler for measurement. Diagnostic imaging by ultrasound or other imaging modality, should be performed when concurrent umbilical or epigastric hernia or other cause of the patient’s symptoms cannot be excluded. (2) Physiotherapy is the firsthand treatment for diastasis of the rectus abdominis muscle. Surgery should only be considered in diastasis of the rectus abdominis muscle patients with functional impairment, and not until the patient has undergone a standardized 6-month abdominal core training program. (3) The largest width of the diastasis should be at least 5 cm before surgical treatment is considered. In case of pronounced abdominal bulging or concomitant ventral hernia, surgery may be considered in patients with a smaller diastasis. (4) When surgery is undertaken, at least 2 years should have elapsed since last childbirth and future pregnancy should not be planned. (5) Plication of the linea alba is the firsthand surgical technique. Other techniques may be used but have not been found superior.

**Discussion:**

The level of evidence behind these statements varies, but they are intended to lay down a standard strategy for treatment of diastasis of the rectus abdominis muscle and to enable uniformity of management.

## Introduction

Diastasis of the rectus abdominis muscles (DRAM) is defined as increased separation of the medial edges of the two rectus muscles due to stretching and laxity of the linea alba^1^. It is commonly associated with an abdominal bulge without fascial defect. The upper limit of physiological separation of the rectus muscles varies in different studies as does the recommended point of measurement^2,3^.

DRAM is not a hernia and there is no risk of incarceration. The widening and thinning of the linea alba as well as the bulging of the abdominal wall may, however, be associated with increased risk of developing midline herniation such as epigastric and umbilical hernia^4,5^. The increased inter-rectus distance (IRD) in pregnant women represents one aspect of the general physiological relaxation of connective tissues in anticipation of partus. The increase in intra-abdominal pressure also plays a role in the pathophysiology of DRAM^5–9^.

DRAM may cause cosmetic impairment, abdominal and lower back pain, as well as reduced strength of the trunk muscles^6,7^. It has been suggested that it is not the diastasis *per se* but rather the bulging or protrusion of the entire abdominal wall that causes functional disability^8,9^.

Core training improves physical function and quality of life^7^. Its effect on reducing the diastasis as such, however, is not well-established^10–12^.

The role of surgery in the treatment of DRAM is controversial. Most operations are still done for aesthetic reasons and as part of abdominoplasty^10,13–15^. Since surgery solely aiming at correction of cosmetic defects is currently not supported by the Swedish public healthcare system, most diastasis patients are operated in private hospitals. However, functional disability related to DRAM falls under the responsibility of the public healthcare system, and substantial regional differences in access to DRAM surgery have been identified. If we are to provide the treatment necessary on equal terms, we must have Swedish national guidelines on the management of DRAM.

The objective of this document was to define and present recommendations for the management of patients with symptomatic diastasis of the rectus muscles (DRAM) for use as a basis for future guidelines. These recommendations will focus on indications for surgery.

## Methods

In 2017, a group of Swedish specialists in surgery were gathered together by the Swedish Association of Innovative Surgical Technology and the Swedish Surgical Society to discuss and present evidence-based recommendations to be used in future guidelines on the management of DRAM. The group consisted of abdominal surgeons and plastic surgeons with experience in DRAM surgery.

The present recommendations have been developed in collaboration with the Health Technology Assessment Group of the South-East Region of Sweden.

**Fig. 1.**
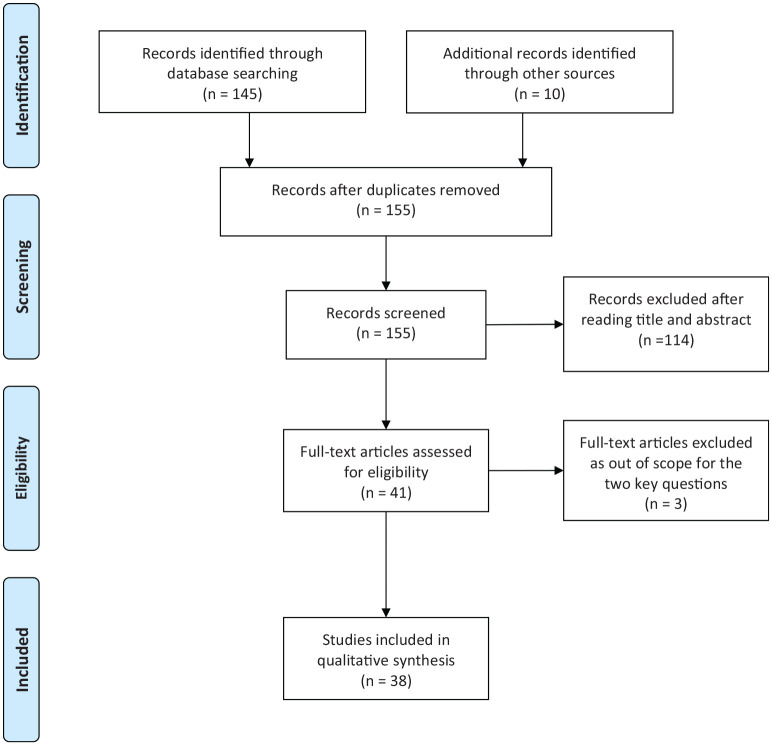
Prisma flow chart.

A search in PubMed was performed by the Health Technology Assessment Group of the South-East Region of Sweden 2019-11-07. A total of 86 references were identified using the following search terms: Diastasis (All Fields) AND recti (All Fields) AND (“therapy”(Subheading) OR “therapy”(All Fields) OR “treatment”(All Fields) OR “therapeutics”(MeSH Terms) OR “therapeutics”(All Fields)). A further 59 references were found using the following search terms: Diastasis (All Fields) OR (divarication (All Fields) AND recti(All Fields)) OR rectus(All Fields)) AND (randomized(All Fields) AND controlled(All Fields)). See prisma flow diagram (Fig. 1).

The recommendation group identified the following *key-questions*:

1. What is the expected outcome of physiotherapy in patients with DRAM?
2. Which patients should be considered for operative correction of DRAM (Indications and contraindications for surgery)?

The levels of evidence and grades of recommendations were rated according to the Oxford Center for Evidence-based Medicine—Levels of Evidence^16^.

## Results

Studies identified in the search are listed in Table 1. Studies exploring the outcome after surgery are listed in Table 2.

**Table 1.** Studies included in the qualitative synthesis.

Author	Year	Study type	No. of patients	Main finding
Effect of physiotherapy				
Mota et al. ^28^	2015	Longitudinal cohort study	84	Abdominal Crunch Exercise produced significant narrowing of the IRD
Gluppe et al. ^29^	2018	RCT	175	No significant effect on IRD of a postpartum training program
Thabet et al. ^12^	2019	RCT	40	A deep core stabilizing program reduces inter-recti distance (IRD) in postpartum women with DRAM
Emanuelsson et al. ^9^	2016	RCT	32	Improved muscular strength, function, and quality of life after a 3 months training program in patients with DRAM
Width of the diastasis				
Mota et al. ^18^	2018	Prospective cohort study	84	Definition of normal IRD during pregnancy and 6 months postpartum
Ranney ^4^	1990	Cross-sectional descriptive study	1763	Classification of DRAM. High prevalence of umbilical hernias in women with DRAM.
Liaw et al. ^20^	2011	Prospective cohort study	40 + 20 controls	No clear relationships found between width of diastasis and abdominal muscle function in postpartum women.
Gunnarsson et al.^21^	2015	Cross-sectional descriptive study	57	A positive correlation existed between abdominal muscle strength and IRD below the umbilicus, but not when IRD was measured above the umbilicus.
Kohler et al. ^30^	2018	Case series	20	Concomitant repair of ventral hernias and DRAM
Olsson et al. ^5^	2019	Prospective cohort study	60	75% of women operated for DRAM had concomitant ventral hernias
Preoperative diagnostic imaging				
Mota et al. ^24^	2012	Test–retest reliability study	24	Ultrasound imaging is a reliable method for measuring the IRD
Keshwani et al. ^23^	2018	Cross-sectional study	32	Ultrasound measurement of IRD in the early postpartum period correlated well to symptoms of DRAM.
Emanuelsson et al.^31^	2014	Cross-sectional study	55	Clinical assessment prior to surgery provides more accurate information than CT scanning in the assessment of ARD width.
Time between last childbirth and operation				
Boissonnault et al. ^25^	1988	Cross-sectional study	71	DRAM most common in third trimester and persists in the immediate postpartum period.
Sperstad et al. ^26^	2016	Prospective cohort study	300	Prevalence of DRAM was 33% 12 months after delivery.
Ranney^4^	1990	Cross-sectional study	1738	Less than 1% of parous women had a “severe” diastasis exceeding 5 cm.
Surgical methods				
Van Uchelen et al.^32^	2001	Cross-sectional study	40	40% recurrence rate after repair of DRAM as part of abdominoplasty.
Bellido Luque et al.^33^	2015	Prospective cohort study	21	No recurrence after totally endoscopic approach to diastasis recti associated with midline hernias (no mesh)
Köhler et al. ^30^	2018	Prospective cohort study	20	No recurrence at 5 months after minimal invasive linea alba reconstruction (MILAR)
Köckerling et al. ^34^	2016	Prospective cohort study	40	No early recurrences after endoscopic-assisted linea alba reconstruction plus mesh augmentation (ELAR plus)
Emanuelsson et al. ^9^	2016	RCT	86	No difference in outcome between retromuscular mesh repair and double-row self-retaining sutures.
Nahas et al. ^35^	2005	Case series	12	No recurrence rate 6–7 years after plication of DRAM.
Olsson et al. ^5^	2019	Prospective cohort study	60	Significant improvement in quality of life and abdominal trunk function after surgical repair of DRAM.

IRD: inter-recti distance; RCT: randomized controlled trials; DRAM: diastasis of the rectus abdominis muscle; ARD: abdominal rectus diastasis; MILAR: minimal invasive linea alba reconstruction; ELAR: endoscopic-assisted linea alba reconstruction plus mesh augmentation.

**Table 2.** DRAM: Outcome of surgery.

Author	Year	Study type	No	Follow-up	Main findings
Emanuelsson et al. ^9^	2016	RTC Retro muscular mesh repair versus double-row self-retaining sutures (Quill)	86	1 year	Improved abdominal wall stability and muscle strength. Improved functional ability and quality of life. No difference between the two groups at 1 year. One early recurrence in the Quill group. Five (6%) patients with encapsulated seroma needing reoperation.
Olsson et al. ^5^	2019	Prospective Cohort study. Women with DRAM and symptoms resistant to training. Open double-row plication of linea alba (Quill)	60	1 year	Surgical reconstruction resulted in improved abdominal trunk function and quality of life (SF-36) at 1 year. No recurrence was noted at one year of follow-up. Postoperative complications (bleeding, wound infection and seroma) was found in eleven patients. Reoperation was required in four patients.
Van Uchelen et al. ^32^	2001	Cross-sectional study	40	32–109 months	40% recurrence rate after suture repair of DRAM in connection with abdominoplasty.
Nahas et al. ^35^	2005	Case series	12	76–84 months	No recurrent diastasis after repair with non-absorbable suture in connection with abdominoplasty
Shirah and Shirah ^36^	2016	Retrospective cohort study Comparing open and laparoscopic mesh repair	216	2 years	Wound infections and seroma more common in the open repair group. No recurrence in any of the two groups after 24 months of follow-up.

DRAM: diastasis of the rectus abdominis muscle.

### Effect of Physiotherapy

Evidence supporting an effect of training programs in the prevention or treatment of DRAM width is generally weak^17^. In a recently published report, however, Thabet and Alshehri(12) showed a significant reduction in IRD after 8 weeks of a “deep core stability exercise program” compared to a control group who underwent a traditional exercise program.

However, there is strong evidence in favor of a positive effect of core training on abdominal muscle strength and function. Emanuelsson and his colleagues^7,9^ found that a 3-month training program improved objectively measured muscular strength. In their study, significant functional improvement reported in a validated questionnaire, the Ventral Hernia Pain Questionnaire (VHPQ), was seen. The training program also had a positive effect on quality of life. However, neither compliance with the training program nor the long-term impact on functional outcome was reported^7,9^.

Although there are few studies showing a long-lasting effect of core training on symptoms related to DRAM, there is widespread agreement that non-invasive treatment is the firsthand choice for a condition that is essentially related to abdominal trunk function and not associated with any potentially severe event requiring surgical treatment^10^.

#### Level of evidence 2C: Outcome studies.

Recommendation (Grade C): The firsthand treatment for DRAM is core training. Surgery should not be considered until the patient has undergone a training program for at least 6 months.

### Width of the Diastasis

There are several classifications of DRAM based on the width of the diastasis at different points of measurement.

In a longitudinal study of 84 primiparous women using ultrasound, Mota et al. (18) found the upper limits of IRD to be 28 mm above the umbilicus and 21 mm below the umbilicus at 6 months postpartum.

The following classification of DRAM was proposed by Ranney in 1990: *mild diastasis* *<* *3* *cm, moderate 3–5* *cm*, and *severe diastasis* *>* *5* *cm*^4^. A classification of rectus diastasis using five points of measurements along the midline has recently been proposed by Reinpold et al.^19^.

There is no clear association between the width of the diastasis and abdominal muscle function in postpartum women^20^. Gunnarsson et al.^21^ found a strong correlation between muscle strength and rectus diastasis width below the umbilicus. The correlation, however, was only statistically significant when using intraoperative measurement of the diastasis. In their study, no correlation was found between muscle strength and IRD above the umbilicus.

The presence of an associated ventral hernia may be an indication for surgery, regardless of the size of a concomitant diastasis^2,4^. The surgical procedure should focus on the repair of the hernia, but may also include closure of the diastasis. In a cohort study, Olsson et al. (5) showed a perioperative incidence of concomitant epigastric and/or umbilical hernia of 75%.

#### Level of evidence 4: consensus agreement.

Recommendation (Grade D): The largest width of the diastasis should be at least 5 cm (“severe diastasis”) for surgery to be considered. Surgical repair of the diastasis is recommended in patients with a symptomatic ventral hernia irrespective of the width of the diastasis. In the case of pronounced abdominal bulging or when performing trials, surgery on patients with a diastasis exceeding 3 cm may be considered.

### Preoperative Diagnostic Imaging

There is no international consensus on which method of measurement should be used to measure the inter-recti distance in DRAM^22–24^. In a systematic review of different methods, van de Water and Benjamin concluded that both calipers and ultrasound are adequate tools to assess DRAM, although ultrasound imaging is most widely used^22^. The advantage of ultrasound is its ability to detect any associated hernia, which may strengthen the indication for surgical repair.

#### Level of evidence 2C: outcome studies.

Recommendation (Grade C): Diagnostic imaging by ultrasound should be done prior to surgery in cases where concurrent umbilical or epigastric hernia is suspected or it is not possible to determine the width of the diastasis at clinical examination. Computed tomography (CT) scan may be used to rule out other pathology.

### Time Between Last Childbirth and Surgery

Most women develop DRAM during the last trimester, and this persists into the immediate postpartum period^25^. Separation of the rectus muscles gradually decreases with time after delivery. In a cohort study of 300 women, Sperstad et al.^26^ found a 60% prevalence of DRAM 6 weeks postpartum gradually decreasing to 33% 12 months after delivery. In their study, measuring inter-recti distance using a finger-width method, no woman was found to have severe diastasis (exceeding 5–6 cm) and only two women had a diastasis that could be classified as “moderate.” This finding corresponds well with figures reported by Ranney 1990, that only 0.7% of 1738 parous women had a diastasis exceeding 5 cm^4^.

#### Level of evidence 4: Consensus agreement.

Recommendation (Grade D): At least 2 years should have elapsed since last childbirth before considering surgery, and pregnancy thereafter should not be planned.

### Surgical Methods

Different techniques for surgical treatment of DRAM have been described.

The two predominating questions are whether to use an open or laparoscopic technique and whether to reinforce the linea alba with a mesh^27^. In a recent review, Mommers et al.^10^ reported that 85% of repairs use an open procedure. In open surgery, the incision is either midline or transverse in the lower half of the abdomen. The best cosmetic outcome is generally considered to be achieved through a transverse incision in the lower half of the abdomen combined with abdominoplasty, but this is a longer procedure and requires more surgical experience than simple plication of the linea alba via a midline incision. Most studies on surgical technique are retrospective case studies with low to moderate quality (^10^; Table 1).

#### Outcome and complications.

Recurrence rates vary from 0% to 40% in the long-term follow-up studies ^32,35^. There are only a few randomized controlled trials (RCT) comparing the outcomes of different techniques. In an RCT including 56 patients comparing a Quill suture technique with mesh reinforcement, Emanuelsson et al. ^9^ found no difference between groups in recurrence rate or functional results 1 year after surgery and at a 5-year follow-up (data submitted for publication). In a retrospective study comparing open and laparoscopic mesh repair, Shirah and Shirah ^36^ found no differences in postoperative complication or recurrence rates 2 years after surgery. In their cohort, the mean inter-recti distance was 10 cm in both groups, which could question the external validity of the study.

Postoperative complications include formation of seroma, wound infection, and chronic pain. Persistent loss of sensation due to nerve injury has been reported after procedures involving abdominoplasty ^37^. Patient satisfaction is generally reported to be acceptable, but few studies have used a validated instrument for the evaluation of patient-reported outcome (PRO). Olsson et al. (5) showed significant improvements in self-reported functionality and quality of life using two validated forms, the Disability Rating Index (DRI) and the short form-36 (SF-36) quality-of-life assessment form, 1 year after surgery. Emanuelsson et al. ^9^ used a validated questionnaire for pain assessment (VHPQ) and reported significant improvement in all modalities at follow-up. Furthermore, they found a significant improvement in quality of life (SF-36) 1 year postoperatively with no difference seen between the two study arms.

#### Level of evidence 1B: RCTs of good quality.

Recommendation (Grade B): Plication of the linea alba is the gold standard and firsthand surgical technique. Other techniques may be practiced locally but have not been found superior in terms of abdominal trunk function.

#### Quality assessment.

As there is very limited evidence regarding the benefit of surgery for DRAM, there is a need for standardized validated tools to assess function and patient-reported symptoms pre- and postoperatively, as well as a dedicated register for postoperative complications and recurrence after surgery for DRAM.

#### Level of evidence 4: consensus agreement.

Recommendation (Grade D): There should be standardized follow-up and quality assessment of surgical treatment for DRAM, preferably using a nationwide patient register.

## Discussion

There is still no international consensus on the treatment of diastasis recti. The role of surgery is controversial and international guidelines are lacking ^10^. Most DRAM procedures are performed during abdominoplasty or for cosmetic reasons and consequently not covered by the public healthcare system in Sweden. Growing evidence that diastasis may also be associated with substantial functional impairment with negative impact on the woman’s quality of life has led to an increase in the demand for national guidelines, and this has recently received considerable attention in the Swedish media. Management of DRAM varies substantially between regions in Sweden. The focus of the present paper concerns indications for surgical correction of DRAM aiming to provide recommendations that may be implemented in future national guidelines.

The results of the present investigation confirm several previous reports that there are few evidence-based recommendations for the management of DRAM. Most reports are of low to moderate scientific quality. Comparison between studies is difficult due to lack of consensus on cut-off points and measurement tools that should be used in the definition of DRAM. Furthermore, studies on long-term outcome comparing core training and surgery are lacking. Most studies have been done on postpartum women and may not necessarily apply to men or nulliparous women.

Core training programs may only partially reduce the inter-recti distance in women with established DRAM, with limited effect on cosmetic outcome. However, there is evidence that core training may lead to considerable functional improvement and increase in abdominal trunk muscle strength. Emanuelsson et al. (9, 13), however, reported that patient satisfaction was lower after a 3-month training program compared to patients who were operated. We need to define how core training should be performed and after how long its effect should be evaluated.

We recommend that all patients should undergo a core training program for a period of at least 6 months before being considered for surgical correction of the diastasis.

At present, there are few reports in the literature regarding the correlation between the width of the diastasis and physical symptoms. Gunnarsson et al. reported a negative correlation between objectively measured muscle strength and inter-recti distance. This, however, was only statistically significant for diastases below the umbilicus. The authors concluded that diastasis width should be used as one of the criteria for surgical treatment. There is an urgent need for studies on the relationship between the degree of diastasis (both width and length), measured in a standardized manner, and physical symptoms.

We recommend that an inter-recti distance of at least 5 cm measured at the widest point along the linea alba should be used as a criterion for surgical treatment. This corresponds to “severe diastasis” according to the classification suggested by Ranney^4^. An IRD less than 5 cm may be accepted for surgery when there is excessive bulging of the abdominal wall or in the presence of an epigastric or umbilical hernia. A diastasis less than 5 cm may also be accepted as a criterion in clinical trials.

It is essential that the IRD is measured and recorded in a standardized manner with the patient in a relaxed supine position. Ultrasound is the most commonly used method in current research and has the advantage of being able to detect a small ventral herniation. The use of calipers or a ruler is a validated alternative. Reinpold et al. (19) in a recent review recommended a classification of DRAM based on five points for IRD measurement as well as the length of the diastasis. An instrument for measuring symptom load including abdominal bulging would be valuable.

When deciding on the method of repair, it must be remembered that DRAM is not a hernia and therefore carries no potential risk of strangulation.

DRAM repair is often combined with abdominoplasty in order to improve the cosmetic outcome. This procedure is technically more difficult with a potentially higher rate of long-term complications^6^. Such cases should be referred to centers with experience in these procedures. Based on the findings of Emanuelsson et al. ^9^ that mesh reinforcement has no advantage over Quill repair at 1 year, we recommend that plication of the linea alba with double-row sutures via a midline incision be the standard procedure in a general surgical setting. In a recently published cohort study on 60 postpartum women who had not responded to training, Olsson et al. (5) showed significant improvements in abdominal trunk function and quality of life (SF-36) 1 year after surgery using double-row plication of the linea alba without mesh.

Novel minimally invasive endoscopic methods, including mesh reinforcement, have been described for the repair of DRAM with associated ventral hernia^33,34,38^. Comparative studies and long-term results are not yet available.

PRO, that is, functional results and quality of life including satisfactory cosmetic result should be included in future studies^10^.

There is an urgent need for further studies comparing different repair techniques with PRO as primary outcome. Qualitative methods, focusing on the patient’s perspective and expectations, may be of value in this respect.

As the evidence in favor of surgery for DRAM is very limited, there is a need for standardized assessment of short- and long-term outcomes after DRAM repair. If there is to be support for surgical repair of a condition that is not associated with mortality or unequivocally defined morbidity in a publicly financed healthcare system, outcomes must be meticulously assessed and transparently presented to the healthcare provider.

## Summary and Conclusion

This consensus report, based on current literature, was produced by a working group under the auspices of the Swedish Surgical Society. It provides recommendations that may be used in future national guidelines on the management of DRAM.

Rectus diastasis is associated with both cosmetic and functional disability, especially in women after childbirth. The level of evidence for management of rectus diastasis is generally low and great regional differences in treatment exist in Sweden. Training programs specifically targeting DRAM lead to significant increases in physical function though cosmetic improvement is limited.

The indication for surgical treatment of DRAM in the absence of associated ventral hernia is still controversial. Several methods of repair have been described including plication with or without mesh reinforcement. Open repair techniques dominate but new minimally invasive endoscopic or endoscopic-assisted methods have been described with promising short-term results.
